# Comparing SurePath, ThinPrep, and conventional cytology as primary test method: SurePath is associated with increased CIN II^+^ detection rates

**DOI:** 10.1007/s10552-015-0678-1

**Published:** 2015-10-12

**Authors:** Kirsten Rozemeijer, Corine Penning, Albert G. Siebers, Steffie K. Naber, Suzette M. Matthijsse, Marjolein van Ballegooijen, Folkert J. van Kemenade, Inge M. C. M. de Kok

**Affiliations:** Department of Public Health, Erasmus MC, University Medical Center, Room Na-2223, PO Box 2040, 3000 CA Rotterdam, The Netherlands; Department of Pathology, Radboud University Medical Center, PO Box 9101, 6500 HB Nijmegen, The Netherlands; PALGA, The Nationwide Network and Registry of Histo- and Cytopathology in The Netherlands, Randhoeve 231A, 3995 GA Houten, The Netherlands; Department of Pathology, Erasmus MC, University Medical Center, PO Box 2040, 3000 CA Rotterdam, The Netherlands

**Keywords:** Cervical intraepithelial neoplasia, Liquid-based cytology, SurePath, ThinPrep, Conventional cytology, Screening

## Abstract

**Purpose:**

Within the last decade, SurePath and ThinPrep [both liquid-based cytology (LBC) tests] have replaced conventional cytology (CC) as primary test method in cervical cancer screening programs of multiple countries. The aim of our study was to examine the effect in the Dutch screening program.

**Methods:**

All primary smears taken within this program from 2000 to 2011 were analyzed using the nationwide registry of histo- and cytopathology (PALGA) with a follow-up until March 2013. The percentage of smears classified as borderline/mildly dyskaryotic (BMD) and >BMD as well as CIN and cervical cancer detection rates were compared between SurePath and ThinPrep versus CC by logistic regression analyses (adjusted for age, screen region, socioeconomic status, and calendar time).

**Results:**

We included 3,118,685 CC, 1,313,731 SurePath, and 1,584,587 ThinPrep smears. Using SurePath resulted in an increased rate of primary smears classified as >BMD [odds ratio (OR) = 1.12 (95% confidence interval (CI) 1.09–1.16)]. CIN I and II^+^ detection rates increased by 14 % [OR = 1.14 (95% CI 1.08–1.20)] and 8 % [OR = 1.08 (95% CI 1.05–1.12)]. Cervical cancer detection rates were unaffected. Implementing ThinPrep did not result in major alterations of the cytological classification of smears, and it did not affect CIN detection rates. While not significant, cervical cancer detection rates were lower [OR = 0.87 (95% CI 0.75–1.01)].

**Conclusions:**

The impact of replacing CC by LBC as primary test method depends on the type of LBC test used. Only the use of SurePath was associated with increased CIN II^+^ detection, although it simultaneously increased the detection of CIN I.

## Introduction

**Fig. 1:**
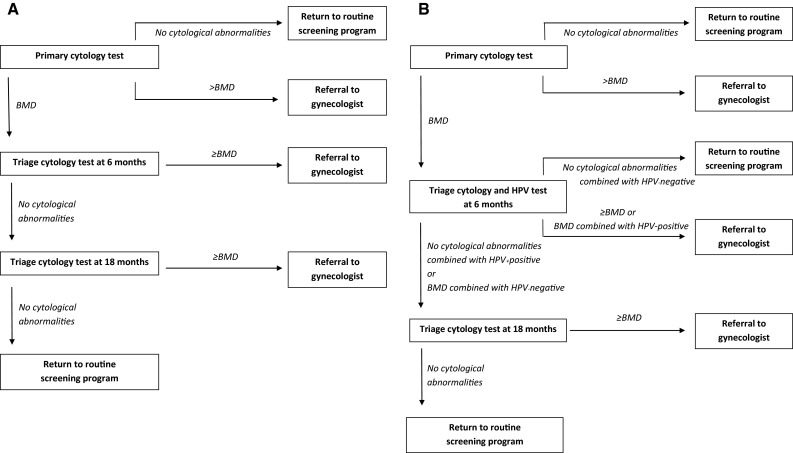
Triage protocol consisting of triage cytology without HPV testing (**a**) and with HPV testing (**b**). *HPV* human papillomavirus, *BMD* borderline and mildly dyskaryotic smears

Since the 1980s, a national cervical cancer screening program exists in the Netherlands. From 1996 onwards, women are invited every five years from ages 30 to 60 years. The screening strategy consists of primary cytology screening with triage by repeat cytology or triage by a combination of repeat cytology and human papillomavirus (HPV) testing (Fig. 1). Despite its limited sensitivity [1], the conventional cytology test has long been used as primary test method.

Within the last 10–15 years, conventional cytology has been replaced by liquid-based cytology (LBC) tests SurePath or ThinPrep in most of Dutch laboratories processing primary screening tests. Conventional cytology and both LBC systems share the same method of sampling cells from the cervix (i.e., scraping off cells with a brush or similar device from the histological transition zone). LBC differs from conventional cytology with respect to the transfer of cells from brush to slide: With conventional cytology, cells are directly smeared on a slide, while with LBC, the brush is first rinsed into a vial with a preservative fluid and then transported to a laboratory [2]. In the laboratory, an uniform layer of cells is prepared on the slide [3, 4]. It is thought that this method of cell transfer (which differs between SurePath and ThinPrep) results in a better representation of the entire sample as compared to conventional cytology [5]. A review which evaluated the applicability of LBC in the Dutch cervical cancer screening program concluded that further research was needed to determine the applicability of SurePath. Furthermore, they recommended to further analyze the costs and benefits of ThinPrep before deciding whether or not to implement this method [6]. Yet, public health authorities in the Netherlands permitted use of both LBC systems based on perceived advantages such as: ease of processing, reduction in unsatisfactory slides [7–10], and time needed to read the slides [10–13]. Finally, the use of LBC allowed for easier application of HPV co-testing [14, 15].

The use of conventional cytology as primary test method has also been replaced by the use of SurePath and/or ThinPrep in many other countries with and without organized cervical cancer screening programs, such as Denmark, the UK, and the USA [16, 17]. It is believed that the sensitivity of LBC for detecting cervical intraepithelial neoplasia (CIN) II^+^ lesions is similar to that of conventional cytology [18, 19]. However, when stratifying for the type of LBC test used, many studies have been published comparing CIN detection between ThinPrep and conventional cytology [2, 5, 11, 20–22], while only two studies have compared CIN detection between SurePath and conventional cytology [10, 23]. Moreover, no studies have been published comparing CIN detection rates between the three types of cytology tests. As the outcome of all cervix uteri cytological and histological tests taken within the Dutch screening program were available (i.e., are registered in the Dutch Pathology Register (i.e., in PALGA) [24]) and we were able to deduce which type of primary cytology test had been used, we assessed whether differences in CIN detection rates were present when screened by SurePath or ThinPrep as compared to conventional cytology. In addition, we assessed the effect on cervical cancer detection rates and on the classification of smears.

## Methods

Information on all cervix uteri cytological and histological tests in the Netherlands registered from January 2000 until March 2013 was retrieved from PALGA. Women are identified through their birth date and the first eight letters of their (maiden) family name. This identification code enables linkage of tests belonging to the same woman, allowing us to follow individual screening histories. We identified primary smears (i.e., first smear of an episode) taken within the national cervical cancer screening program between January 2000 and December 2011. A minimum duration of 15-month follow-up was ensured as data until March 2013 were available. Histologically confirmed CIN lesions and cervical cancer cases were identified by selecting all PALGA records that included corresponding pathology codes. Detection of these conditions was assigned to the type of cytology test used. Age was defined as the woman’s age at the time of the primary smear and was categorized as 29–33, 34–38, 39–43, 44–48, 49–53, 54–58, and 59–63 years. As women are invited every 5 years in the year they turn 30, 35,…, 60 years, these age categories reflect different screening rounds. The cervical cancer screening program is organized by five different screening organizations, each accounting for a geographical region (i.e., screen region) (north, southwest, middle west, south, and east). Screen regions were coded corresponding with the place of residence at the time of the primary smear. Socioeconomic status (SES) was defined (low, middle, high) according to the status score, which is an ecological variable based on the four-digit postal code of the woman’s place of residence at the time of the primary test [25]. Status scores per four-digit postal code were provided by the Netherlands Institute for Social Research, www.scp.nl based on (1) mean income, (2) percentage of households with a low income, (3) percentage of households with, on average, a low education, and (4) unemployment rate in 2010. These variables were merged into one score (i.e., status score) by a principal component analysis. Low SES corresponded with a status score lower than −1 (i.e., average status score minus standard deviation), intermediate SES with a score of ≥−1 and ≤1, and high SES with a score higher than 1 (i.e., average status score plus standard deviation).

In PALGA, the type of cytology testing is not routinely registered. Therefore, the date of conversion was retrieved from the laboratories fixed to one of the quarters per year, since most laboratories had a phase-in–phase-out transition period of 2–4 months. This information was linked to PALGA as a proxy for which type of primary cytology test was used (i.e., in the Netherlands, laboratories supply the tools for cytology and thus determine the type of cytology test that is used by the general practitioner).

### Type of cytology testing

With conventional cytology, cervical specimen is collected (i.e., no data were available on the type of device or brush used), and cells are directly smeared from the sampling device on the slide. With SurePath, cervical specimen is collected using a broom-like device with detachable head. The detachable head is placed in a vial with an ethanol-based preservative fluid. At the laboratory, the fluid and cells are centrifuged to isolate the cells from the fluid. The cells are resuspended in a sucrose density gradient followed by slide transfer using gravity for adherence. With ThinPrep, cervical specimen is collected using a Cervix Brush, and the brush is rinsed in a vial with a methanol-based preservative fluid. Cells are released by pushing the brush to the bottom, forcing the bristles apart, and swirling the brush into the fluid. Subsequently, the brush is discarded. At the laboratory, cells are isolated from the fluid by vacuum filtration and are transferred to the slide using air pressure for adherence [26].

###  Statistical analyses

Since LBC was implemented per laboratory at different points in time, calendar time is expected to differ between the three types of cytology tests. The demographic characteristics of attending women (i.e., age, screen region, and SES) also differ between laboratories; hence, we expected that they also differ between the cytology tests. As age, SES, screen region, and calendar time are all associated with CIN and/or cervical cancer [27, 28], they are all potential confounding factors. We used a Pearson’s Chi-squared test to assess whether their distributions differed between the types of cytological tests. Thus, we tested whether they were confounders or not. A *p* value of less than 0.05 was considered to be statistically significant.

We performed logistic regression analyses to examine whether CIN and cervical cancer detection rates differed between the types of cytological tests, adjusted for confounding factors. Moreover, we assessed how these overall changes in CIN and cervical cancer detection rates, if present, were composed. First, we examined whether the rate of primary smears classified as borderline/mildly dyskaryotic (BMD) differed between the types of cytological tests. Second, we assessed whether CIN and cervical cancer detection rates in women with a BMD smear were different between the types of tests, which would indicate that the positive predictive value (PPV) of a primary BMD smear differed. Third, we combined these two steps to examine whether the tests differed in the fraction of primary smears both classified as BMD and resulting in the detection of a CIN or cervical cancer. By performing the same analyses for having a >BMD smear, we could assess whether potential differences in CIN and cervical cancer rates were (mainly) caused by differences in the triage (i.e., those with a primary BMD smear) or direct referral pathway (i.e., those with a primary >BMD smear). Finally, we assessed the overall difference in CIN and cervical cancer detection rates, regardless of the cytological result.

Missing values were imputed with 10 multiple imputations for confounding factors. The odds ratio (OR) was interpreted as relative risk if the prevalence of the outcome (i.e., BMD, >BMD, CIN I, CIN II, CIN III, cervical cancer, or CIN II^+^) was <10 % in the respective logistic regression model analysis [29]. The software program SPSS (version 20) was used to perform the statistical analyses.

## Results

**Fig. 2:**
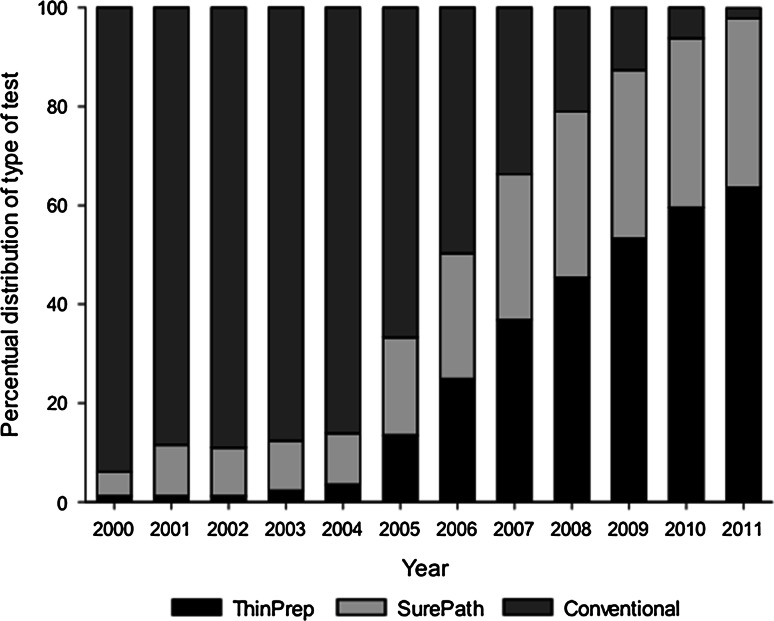
Distribution of the types of cytological tests used within the Dutch screening program. The total number of primary smears where the type of cytological test was known varied from 441,663 in 2000 to 541,587 in 2007

We included 3,118,685 primary conventional cytology smears, 1,313,731 primary SurePath smears, and 1,584,587 primary ThinPrep smears in our analyses. The distribution of calendar time significantly differed between the methods of cytology testing (*p* < 0.001). In 2000, 94 % of the primary cytology tests performed within the Dutch screening program consisted of conventional cytology, while in 2011 this percentage has dropped to 2 % (Fig. 2). The distribution of age, SES, and screen region also significantly differed between the methods of cytology testing (Table 1). For instance, most conventional cytology tests were performed in screen region 4 (28 %), while most SurePath and ThinPrep tests were performed in screen regions 1 (38.4 %) and 2 (34.0 %), respectively. Thus, calendar time, age, SES, and screen region were all considered confounding factors and missing values were imputed for 1.6 % of the primary smears.

**Table 1:** Population characteristics

	Conventional	SurePath	ThinPrep	*p* value
N	3,118,685	1,313,731	1,584,587	
Screen region				<0.001
1, *n* (%)	430,548 (13.8)	503,967 (38.4)	352,790 (22.3)	
2, *n* (%)	822,189 (26.4)	178,844 (13.6)	538,890 (34.0)	
3, *n* (%)	482,137 (15.5)	311,276 (23.7)	296,609 (18.7)	
4, *n* (%)	872,931 (28.0)	294,939 (22.5)	206,098 (13.0)	
5, *n* (%)	501,852 (16.1)	24,471 (1.9)	187,279 (11.8)	
Unknown, *n* (%)	9,028 (0.3)	234 (0.0)	2,921 (0.2)	
SES				<0.001
Low, *n* (%)	257,544 (8.3)	156,058 (11.9)	107,983 (6.8)	
Middle, *n* (%)	2,574,027 (82.5)	1,045,158 (79.6)	1,331,613 (84.0)	
High, *n* (%)	239,623 (7.7)	87,591 (6.7)	132,439 (8.4)	
Unknown, *n* (%)	47,491 (1.5)	24,924 (1.9)	12,552 (0.8)	
Age				<0.001
29–33 years, *n* (%)	428,600 (13.7)	170,699 (13.0)	195,935 (12.4)	
34–38 years, *n* (%)	522,173 (16.7)	191,193 (14.6)	220,462 (13.9)	
39–43 years, *n* (%)	533,438 (17.1)	222,906 (17.0)	271,924 (17.2)	
44–48 years, *n* (%)	496,856 (15.9)	219,118 (16.7)	265,672 (16.8)	
49–53 years, *n* (%)	446,596 (14.3)	195,127 (14.9)	243,354 (15.4)	
54–58 years, *n* (%)	388,637 (12.5)	171,194 (13.0)	207,405 (13.1)	
59–63 years, *n* (%)	302,385 (9.7)	143,494 (10.9)	179,835 (11.3)	

The distributions of factors associated with CIN detection rates between the three primary test methods are given. If a distribution differs significantly between the primary tests (which is tested with a Pearson’s Chi-square test), the variable is considered to be a confounding factor

### The effect of SurePath versus conventional cytology, adjusted for confounding factors

When comparing using SurePath with using conventional cytology as primary test method, 4 % fewer primary smears were classified as BMD [OR of 0.96 (95% confidence interval (CI) 0.94–0.97)], while a BMD smear more often led to a CIN I [OR of 1.26 (95% CI 1.18–1.34)] or CIN II diagnosis [OR of 1.16 (95% CI 1.08–1.25)]. Combined this led to a 20 % [OR of 1.20 (95% CI 1.13–1.27)] and 14 % [OR of 1.14 (95% CI 1.07–1.22)] increase in the fraction of primary smears both classified as BMD and resulting in the detection of a CIN I or CIN II lesion (Table 2, see for the unadjusted results the Appendix).

**Table 2:** Logistic regression analyses on the classification of smears and histological outcomes when tested by SurePath or Thinprep versus conventional cytology, adjusted for age, SES, screen region, and calendar time

Outcome	SurePath	ThinPrep
BMD^a^	0.96 (0.94– 0.97)	1.02 (1.01– 1.04)
PPV of a primary BMD smear on histological outcomes^b^		
CIN I	1.26 (1.18– 1.34)	1.03 (0.97–1.10)
CIN II	1.16 (1.08– 1.25)	1.04 (0.97–1.12)
CIN III	0.95 (0.88–1.02)	0.87 (0.81– 0.94)
Cervical cancer	0.74 (0.49–1.12)	0.62 (0.41– 0.92)
Fraction of primary smears both classified as BMD and resulting in the detection of the following histological outcomes^c^		
CIN I^a^	1.20 (1.13– 1.27)	1.06 (0.99–1.12)
CIN II^a^	1.14 (1.07– 1.22)	1.08 (1.00– 1.15)
CIN III^a^	0.99 (0.92–1.06)	0.93 (0.87–1.00)
Cervical Cancer^a^	0.77 (0.51–1.15)	0.66 (0.43–1.00)
>BMD^a^	1.12 (1.09– 1.16)	0.96 (0.93– 0.99)
PPV of a primary >BMD smear on histological outcomes^b^		
CIN I	0.92 (0.83–1.03)	0.86 (0.77– 0.97)
CIN II	1.06 (0.98–1.15)	1.08 (0.99–1.17)
CIN III	0.97 (0.91–1.03)	1.06 (0.99–1.13)
Cervical cancer	0.94 (0.80–1.10)	0.98 (0.83–1.15)
Fraction of primary smears both classified as >BMD and resulting in the detection of the following histological outcomes^d^		
CIN I^a^	1.05 (0.94–1.16)	0.83 (0.74– 0.92)
CIN II^a^	1.17 (1.09– 1.27)	1.02 (0.94–1.10)
CIN III^a^	1.10 (1.06– 1.15)	1.00 (0.96–1.04)
Cervical cancer^a^	1.07 (0.91–1.24)	0.93 (0.79–1.09)
Overall histological outcomes		
CIN I^a^	1.14 (1.08– 1.20)	0.98 (0.93–1.04)
CIN II^a^	1.14 (1.09– 1.20)	1.04 (0.99–1.10)
CIN III^a^	1.06 (1.02– 1.10)	0.98 (0.94–1.01)
Cervical cancer^a^	0.99 (0.86–1.14)	0.87 (0.75–1.01)

Odds ratios with a 95% confidence interval are given. This table shows how the overall changes in CIN and cervical cancer detection rates, if present, are composed. The differences in the odds of primary smears classified as BMD combined with the differences in the odds of the PPV of a BMD smear led to differences in the fraction of primary smears both classified as BMD and resulting in the detection of a CIN or cervical cancer. By performing the same analyses for having a >BMD smear, we could assess whether potential differences in CIN and cervical cancer rates were (mainly) caused by differences in the triage (i.e., those with a primary BMD smear) or direct referral pathway (i.e., those with a primary >BMD smear). Altogether, this led to differences in odds of overall CIN and cervical cancer detection

Underlined = significant. A *p* value of <0.05 was considered to be statistically significant

*BMD* borderline/mildly dyskaryotic, *PPV p*ositive predictive value

^a^Odds ratio could be interpreted as detection rate ratio because the prevalence of the outcome was <10 %

^b^This can be interpreted as: Does a BMD or >BMD smear more often lead to the following histological outcomes when using SurePath or ThinPrep as compared to conventional cytology

^c^Histological outcomes detected via triage

^d^Histological outcomes detected via direct colposcopy

**Table 3:** Logistic regression analyses on the classification of smears and histological outcomes when tested by SurePath or Thinprep versus conventional cytology, unadjusted and adjusted for confounding factors

	Effect of using either SurePath or ThinPrep instead of conventional cytology as primary test method				
	Unadjusted	Adjusted for year	Adjusted for year and age	Adjusted for year, age, and screen region	Adjusted for year, age, screen region, and SES
*Fraction of primary smears classified as BMD*					
SurePath	1.23 (1.21–1.24)	0.99 (0.97–1.01)	0.99 (0.97–1.00)	0.97 (0.95–0.98)	0.96 (0.94–0.97)
ThinPrep	1.40 (1.39–1.42)	1.05 (1.03–1.07)	1.05 (1.03–1.06)	1.02 (1.00–1.04)	1.02 (1.01–1.04)
*PPV of a primary BMD smear on histological outcomes* ^a^					
CIN I					
SurePath	1.37 (1.30–1.43)	1.23 (1.17–1.31)	1.23 (1.16–1.30)	1.26 (1.19–1.34)	1.26 (1.18–1.34)
ThinPrep	1.16 (1.11–1.21)	1.03 (0.97–1.09)	1.02 (0.96–1.08)	1.04 (0.97–1.10)	1.03 (0.97–1.10)
CIN II					
SurePath	1.29 (1.22–1.36)	1.17 (1.10–1.25)	1.15 (1.07–1.23)	1.17 (1.09–1.26)	1.16 (1.08–1.25)
ThinPrep	1.18 (1.12–1.24)	1.07 (0.99–1.14)	1.04 (0.97–1.12)	1.04 (0.97–1.12)	1.04 (0.97–1.12)
CIN III					
SurePath	0.94 (0.89–1.00)	0.99 (0.92–1.06)	0.95 (0.89–1.02)	0.94 (0.88–1.02)	0.95 (0.88–1.02)
ThinPrep	0.87 (0.82–0.92)	0.93 (0.87–1.00)	0.91 (0.84–0.98)	0.87 (0.81–0.94)	0.87 (0.81–0.94)
Cervical cancer					
SurePath	0.70 (0.51–0.95)	0.76 (0.52–1.11)	0.76 (0.53–1.11)	0.74 (0.60–0.91)	0.74 (0.49–1.12)
ThinPrep	0.58 (0.43–0.77)	0.65 (0.43–0.96)	0.64 (0.43–0.96)	0.62 (0.50–0.77)	0.62 (0.41–0.92)
*Fraction of primary smears both classified as BMD and resulting in the detection of the following histological outcomes* ^b^					
CIN I					
SurePath	1.62 (1.55–1.69)	1.20 (1.14–1.27)	1.20 (1.14–1.27)	1.22 (1.15–1.29)	1.20 (1.13–1.27)
ThinPrep	1.60 (1.53–1.67)	1.07 (1.01–1.13)	1.07 (1.01–1.14)	1.05 (0.99–1.12)	1.06 (0.99–1.12)
CIN II					
SurePath	1.55 (1.47–1.64)	1.15 (1.08–1.22)	1.15 (1.08–1.22)	1.16 (1.09–1.24)	1.14 (1.07–1.22)
ThinPrep	1.63 (1.55–1.71)	1.11 (1.04–1.18)	1.11 (1.04–1.19)	1.07 (1.00–1.15)	1.08 (1.00–1.15)
CIN III					
SurePath	1.16 (1.10–1.22)	0.97 (0.91–1.04)	0.98 (0.92–1.05)	0.99 (0.93–1.07)	0.99 (0.92–1.06)
ThinPrep	1.22 (1.16–1.28)	0.98 (0.91–1.05)	0.99 (0.92–1.06)	0.93 (0.87–1.00)	0.93 (0.87–1.00)
Cervical cancer					
SurePath	0.85 (0.63–1.16)	0.75 (0.52–1.09)	0.75 (0.52–1.09)	0.77 (0.63–0.95)	0.77 (0.51–1.15)
ThinPrep	0.81 (0.60–1.08)	0.68 (0.46–1.01)	0.68 (0.46–1.01)	0.66 (0.53–0.81)	0.66 (0.43–1.00)
*Fraction of primary smears classified as >BMD*					
SurePath	1.21 (1.18–1.24)	1.06 (1.03–1.09)	1.07 (1.04–1.10)	1.15 (1.11–1.18)	1.12 (1.09–1.16)
ThinPrep	1.11 (1.09–1.14)	0.93 (0.90–0.96)	0.93 (0.90–0.96)	0.95 (0.92–0.99)	0.96 (0.93–0.99)
*PPV of a primary >BMD smear on histological outcomes* ^a^					
CIN I					
SurePath	1.03 (0.95–1.12)	0.94 (0.85–1.05)	0.93 (0.84–1.04)	0.92 (0.83–1.03)	0.92 (0.83–1.03)
ThinPrep	0.98 (0.90–1.06)	0.87 (0.77–0.97)	0.87 (0.78–0.98)	0.86 (0.77–0.97)	0.86 (0.77–0.97)
CIN II					
SurePath	1.14 (1.07–1.21)	1.04 (0.96–1.12)	1.04 (0.97–1.13)	1.06 (0.98–1.15)	1.06 (0.98–1.15)
ThinPrep	1.23 (1.16–1.30)	1.09 (1.00–1.18)	1.08 (1.00–1.17)	1.08 (0.99–1.18)	1.08 (0.99–1.17)
CIN III					
SurePath	0.99 (0.94–1.03)	0.97 (0.92–1.03)	0.99 (0.94–1.05)	0.97 (0.91–1.03)	0.97 (0.91–1.03)
ThinPrep	1.13 (1.08–1.19)	1.12 (1.05–1.19)	1.09 (1.03–1.17)	1.06 (0.99–1.13)	1.06 (0.99–1.13)
Cervical cancer					
SurePath	0.96 (0.85–1.08)	0.99 (0.86–1.15)	0.98 (0.85–1.13)	0.93 (0.80–1.09)	0.94 (0.80–1.10)
ThinPrep	0.95 (0.84–1.06)	0.99 (0.85–1.16)	1.00 (0.85–1.17)	0.98 (0.83–1.15)	0.98 (0.83–1.15)
*Fraction of primary smears both classified as >BMD and resulting in the detection of the following histological outcomes* ^c^					
CIN I					
SurePath	1.25 (1.15–1.35)	1.01 (0.91–1.11)	1.01 (0.91. 1.11)	1.07 (0.96–1.19)	1.05 (0.94–1.16)
ThinPrep	1.09 (1.01–1.18)	0.81 (0.73–0.90)	0.82 (0.73–0.91)	0.82 (0.74–0.92)	0.83 (0.74–0.92)
CIN II					
SurePath	1.35 (1.27–1.43)	1.10 (1.02–1.18)	1.10 (1.02–1.18)	1.20 (1.11–1.29)	1.17 (1.09–1.27)
ThinPrep	1.32 (1.25–1.39)	0.99 (0.92–1.07)	1.00 (0.93–1.08)	1.02 (0.94–1.10)	1.02 (0.94–1.10)
CIN III					
SurePath	1.20 (1.16–1.24)	1.05 (1.01–1.09)	1.05 (1.01–1.09)	1.12 (1.08–1.17)	1.10 (1.06–1.15)
ThinPrep	1.17 (1.14–1.21)	0.97 (0.93–1.01)	0.98 (0.94–1.02)	1.00 (0.95–1.04)	1.00 (0.96–1.04)
Cervical cancer					
SurePath	1.16 (1.03–1.31)	1.05 (0.91–1.21)	1.05 (0.91–1.21)	1.08 (0.93–1.26)	1.07 (0.91–1.24)
ThinPrep	1.05 (0.94–1.18)	0.92 (0.79–1.08)	0.92 (0.79–1.07)	0.93 (0.79–1.09)	0.93 (0.79–1.09)
*Overall histological outcomes*					
CIN I					
SurePath	1.50 (1.45–1.56)	1.13 (1.08–1.19)	1.13 (1.08–1.19)	1.16 (1.10–1.21)	1.14 (1.08–1.20)
ThinPrep	1.46 (1.40–1.51)	0.99 (0.94–1.04)	0.99 (0.95–1.05)	0.98 (0.93–1.03)	0.98 (0.93–1.04)
CIN II					
SurePath	1.44 (1.39–1.50)	1.11 (1.06–1.16)	1.11 (1.06–1.16)	1.16 (1.11–1.22)	1.14 (1.09–1.20)
ThinPrep	1.47 (1.42–1.53)	1.05 (1.00–1.10)	1.05 (1.00–1.11)	1.04 (0.99–1.10)	1.04 (0.99–1.10)
CIN III					
SurePath	1.18 (1.15–1.21)	1.02 (0.98–1.05)	1.02 (0.99–1.06)	1.08 (1.04–1.12)	1.06 (1.02–1.10)
ThinPrep	1.18 (1.15–1.21)	0.97 (0.93–1.00)	0.98 (0.94–1.01)	0.97 (0.94–1.01)	0.98 (0.94–1.01)
Cervical cancer					
SurePath	1.08 (0.97–1.21)	0.98 (0.86–1.11)	0.97 (0.85–1.11)	1.01 (0.87–1.16)	0.99 (0.86–1.14)
ThinPrep	1.00 (0.90–1.11)	0.87 (0.76–1.01)	0.87 (0.75–1.00)	0.87 (0.75–1.01)	0.87 (0.75–1.01)
CIN II^+^					
SurePath	1.25 (1.22–1.28)	1.04 (1.02–1.07)	1.05 (1.02–1.08)	1.10 (1.07–1.13)	1.08 (1.05–1.12)
ThinPrep	1.26 (1.23–1.29)	0.99 (0.96–1.02)	1.00 (0.97–1.03)	0.99 (0.96–1.02)	0.99 (0.96–1.02)

Odds ratios with a 95% confidence interval are given. This table shows the impact of adjusting for confounding factors

Underlined = significant. A *p* value of <0.05 was considered to be statistically significant

*SES s*ocioeconomic status, *BMD* borderline/mildly dyskaryotic, *PPV p*ositive predictive value

^a^This can be interpreted as: Does a BMD or >BMD smear more often lead to the following histological outcomes when using SurePath or ThinPrep as compared to conventional cytology

^b^Histological outcomes detected via triage

^c^Histological outcomes detected via direct colposcopy

The rate of primary smears classified as >BMD increased by 12 % [OR of 1.12 (95% CI 1.09–1.16)], whereas a smear classified as >BMD led to a similar number of CIN I, CIN II, CIN III, and cervical cancer diagnoses. As a result, the fraction of primary smears both classified as >BMD and resulting in the detection of a CIN II or CIN III lesion increased by 17 % [OR of 1.17 (95% CI 1.09–1.27)] and 10 % [OR of 1.10 (95% CI 1.06–1.15)].

Overall, CIN I, CIN II, and CIN III detection rates increased by 14 % [OR of 1.14 (95% CI 1.08–1.20)], 14 % [OR of 1.14 (95% CI 1.09–1.20)], and 6 % [OR of 1.06 (95% CI 1.02–1.10)], respectively, when using SurePath as compared to using conventional cytology as primary test method. Cervical cancer detection rates were equivocal between both tests [OR of 0.99 (95% CI 0.86–1.14)]. CIN II^+^ detection rates increased by 8 % [OR of 1.08 (95% CI 1.05–1.12)].

### The effect of ThinPrep versus conventional cytology, adjusted for confounding factors

When using ThinPrep as compared to using conventional cytology as primary test method, the rate of primary smears classified as BMD increased by 2 % [OR of 1.02 (95% CI 1.01–1.04)], although a primary smear classified as BMD less often resulted in a CIN III [OR of 0.87 (95% CI 0.81–0.94) or cervical cancer diagnosis [OR of 0.62 (95% CI 0.41–0.92)]. Combined this led to a marginally significant 8 % increase [OR of 1.08 (95% CI 1.00–1.15)] in the fraction of primary smears both classified as BMD and resulting in the detection of a CIN II lesion. The fraction of primary smears both classified as BMD and resulting in the detection of a CIN I lesion nonsignificantly increased [OR of 1.06 (95% CI 0.99–1.12)], while the fraction both classified as BMD and resulting in the detection of a CIN III or cervical cancer nonsignificantly decreased [ORs of 0.93 (95% CI 0.87–1.00) and 0.66 (95% CI 0.43–1.00) respectively] (Table 2, see for the unadjusted results the Appendix).

The rate of primary smears classified as >BMD decreased with 4 % [OR of 0.96 (95% CI 0.93–0.99)]. A primary smear classified as >BMD less often resulted in a CIN I diagnosis [OR of 0.86 (95% CI 0.77–0.97)], although it nonsignificantly resulted in more CIN II and CIN III diagnoses [ORs of 1.08 (95% CI 0.99–1.17) and 1.06 (95% CI 0.99–1.13) respectively]. As a result, the fraction of primary smears both classified as >BMD and resulting in the detection of a CIN I lesion decreased by 17 % (OR of 0.83 [95% CI 0.74–0.92)].

In total, using ThinPrep as primary test method did not have a significant effect on CIN I (OR of 0.98 (95% CI 0.93–1.04)], CIN II [OR of 1.04 (95% CI 0.99–1.10)], CIN III [OR of 0.98 (95% CI 0.94–1.01)] (Table 2), or CIN II^+^ detection rates [OR of 0.99 (95% CI 0.96–1.02)]. Cervical cancer detection rates were nonsignificantly lower [OR of 0.87 (95% CI 0.75–1.01)].

## Discussion

Using SurePath versus conventional cytology as primary test method resulted in a 12 % increase in the rate of primary smears classified as >BMD. The rate of primary smears classified as BMD decreased by 4 % and women with a primary BMD smear were more often diagnosed with CIN I or II. Combined this led to increased fractions of primary smears both classified as BMD and resulting in the detection of a CIN I or CIN II lesion and to increased fractions of primary smears both classified as >BMD and resulting in the detection of a CIN II or CIN III lesion detected. Altogether, the detection of CIN II^+^ increased by 8 % accompanied by a 14 % increase in the detection of CIN I. Cervical cancer rates were unaffected. The comparison of using ThinPrep versus conventional cytology did not result in such findings, although the sensitivity to detect cervical cancers might be lower.

Given the differences in preparation between both LBC methods, it is possible that the sensitivity for CIN II^+^ differs between them as well. For instance, it was shown that the cell yield is larger when the collecting device was retained instead of discarded from the vial with preservative fluid [30, 31], meaning that if the protocol is followed, the cell yield is larger when using SurePath (i.e., collecting device is retained) than when using ThinPrep (i.e., collecting device is discarded). Therefore, the probability of transferring abnormal cells from the cervical specimen (if present) to the slide is probably larger when using SurePath. The study of Rask et al. [16] seems to confirm this, since they found that replacing conventional cytology by SurePath resulted in a significant 31 % increase in cytological abnormalities within 23–29 aged women, while replacing conventional cytology by ThinPrep resulted in a nonsignificant 11 % decrease [16].

Our research demonstrated that CIN II^+^ detection rates are similar between ThinPrep and conventional cytology, which is compatible with results of previous studies. For instance, the observed CIN II^+^ detection rate ratio of 0.99 (95% CI 0.96–1.02) fits with the pooled relative CIN II^+^ sensitivity of 1.03 (0.97–1.09) as reported in the meta-analysis of Arbyn et al. (i.e., our point estimate lies within the 95% CI) [18]. However, that ratio was based on seven studies comparing LBC with conventional cytology of which two did not use ThinPrep as LBC test method. When only focusing on the included ThinPrep studies, we found and calculated (i.e., using data provided in the study) CIN II^+^ detection rate ratios of 1.17 (95% CI 0.87–1.56) [22], 0.97 (95% CI 0.61–1.55) [20], 0.95 (95% CI 0.62–1.48) [5], and 1.09 (95% CI 0.80–1.48) [21], which were all compatible to the detection rates as observed in the present study. The CIN II^+^ detection rate ratio of the fifth included ThinPrep study was not provided nor could be calculated [11]. Furthermore, the largest randomized controlled trial performed so far, including almost 90,000 participants, found a CIN II^+^ detection rate ratio of 1.00 (95% CI 0.84–1.20) [2] which also fits our data. When focusing on studies comparing SurePath with conventional cytology, only one previous study matched our criteria (i.e., providing a CIN II^+^ detection rate ratio, or data needed to calculate it, at a cutoff of ASCUS or BMD) [23]. Again, their data [i.e., a CIN II^+^ detection rate of 1.01 (95% CI 0.76–1.33)] fitted with ours [i.e., a ratio of 1.08 (95% CI 1.05–1.12)] (i.e., our point estimate lies within the reported 95% CI).

It is expected that from 2016 onwards, primary cytology screening will be replaced by primary HPV screening in the Dutch cervical cancer screening program. If high-risk HPV is present, a reflex cytology triage test will be carried out on the same sample followed by another triage test 6 months later, if the reflex cytology triage test shows no abnormalities. If one of these smears is classified as ≥BMD, the woman will be referred to the gynecologist for colposcopy, otherwise she will be referred to routine screening. Whether our results can be extended from a primary screening to a triage population depends on the performance of the cytology tests on (a) fluid remnant after primary HPV testing in HPV-positive women (in case of reflex triage testing) and (b) directly taken material in (previously) HPV-positive women (in case of triage testing at 6 months). Although prior knowledge of the HPV status influences the interpretation of cytological smears [32, 33], we assume this effect to be similar for the three types of cytology tests. If true, we expect the differences in sensitivity between Surepath, ThinPrep, and conventional cytology in a triage population to be equivalent to the differences in a primary screening population. However, this assumption has not been tested yet. In addition, because conventional cytology cannot be performed on fluid remnant after primary HPV testing [3, 18], our results of comparing SurePath and ThinPrep with conventional cytology cannot be extended to reflex triage testing. As data of Cuzick et al. [34] suggested that the performance of HPV assays depends on the type of LBC test used, it is also possible that the performance of LBC tests on fluid remaining after HPV testing depends on the type of HPV assay used. Thus, more research is needed to assess which combination of primary HPV test and secondary reflex LBC test has the highest CIN II^+^ sensitivity.

We were the first who compared CIN and cervical cancer detection rates between Surepath, ThinPrep, and conventional cytology. Furthermore, we included more than 6 million primary smears, and we showed its effect in real practice instead of in a strictly controlled setting.

At the same time, the lack of a more controlled setting is one of the limitations of our study. As ThinPrep and SurePath were used in different women, differences in demographic factors were inevitable. Although we were able to correct for the confounders age, screen region, SES, and calendar time, we were not able to correct for other potential confounding factors such as screening history or compliance with the given advice. Both could have resulted in biased effect estimates if their distribution differed between the types of cytology tests. In addition, no data are present on whether cytology triage testing at 6 months was combined with HPV testing. Because of the possibility of co-testing, it is likely that the use of HPV triage is correlated with the use of primary LBC testing. As it is known that more CIN I and CIN II lesions are detected when cytology triage is combined with HPV [35], it is probable that the increased sensitivity of SurePath to detect CIN I and CIN II was partly caused by the simultaneous use of HPV testing. However, the entire increase in CIN III detection rates when comparing Surepath with conventional cytology, and for a large part also the increase in CIN II detection rates, is caused by an increase in primary smears being classified as >BMD. Therefore, we still believe that SurePath results in increased CIN II^+^ detection rates, although it might be accompanied by a smaller increase in CIN I detection than estimated. Also, we did not have individual data on which type of primary test was used. Therefore, we combined the date of the primary smear and the quarter of the year within which the laboratory introduced the LBC test as proxy for the type of cytology test that was used. This means that primary screening smears taken during this quarter could have been misclassified, resulting in slightly underestimated effects. Another shortcoming of the study was that we were not able to correct for the use of automated reading, although this has only been introduced in relatively few Dutch laboratories. As study results on the effect of automated screening are heterogeneous, it is unknown how this affected our effect estimates. If automated reading does not affect the sensitivity for CIN II^+^, as shown by Klug and Palmer et al. [36, 37], our estimates are not biased. If automated reading results in a decreased sensitivity for CIN II^+^, as shown in the MAVARIC study [38], we might have underestimated the effect of using SurePath and ThinPrep on CIN II^+^ detection rates. If it results in an increased sensitivity, we might have overestimated the effects. At last, we did not correct for possible learning curve effects, since the aim of our study was to examine the effect of using SurePath and ThinPrep in routine practice, which also includes a possible learning effect.

Our results indicate that the widespread use of SurePath as primary test method has led to an increased probability to detect both CIN I and CIN II^+^ lesions. As only a small fraction of CIN I lesions progress to cancer, increased CIN I detection is often regarded as increased overdiagnosis. In contrast, CIN II^+^ lesions are associated with a substantial cancer risk and are therefore often considered as clinically relevant. However, whether the increased probability to detect CIN II^+^ lesions indeed corresponds with an increased sensitivity for progressive lesions remains to be investigated. If this is the case, using SurePath would in due time result in a decrease in the incidence and mortality of cervical cancer, thereby increasing the health benefits of the screening program. If not, it would only lead to increased burden and harms through overdiagnosis (and treatment) of regressive CIN lesions. The widespread use of ThinPrep as primary test method did not lead to changes in CIN II^+^ detection rates, although cervical cancer detection was nonsignificantly lower. Whether these results imply a decreased sensitivity for progressive CIN II^+^ lesions is unknown. For evidence as to whether the detection of progressive CIN II^+^ lesions is higher with any of the LBC systems than with conventional cytology, cervical interval carcinoma rates have to be compared.

## Appendix

See Table 3.
